# White matter damage in maintenance hemodialysis patients: a diffusion tensor imaging study

**DOI:** 10.1186/s12882-017-0628-0

**Published:** 2017-07-05

**Authors:** David A. Drew, Bang-Bon Koo, Rafeeque Bhadelia, Daniel E. Weiner, Sarah Duncan, Maria Mendoza-De la Garza, Aditi Gupta, Hocine Tighiouart, Tammy Scott, Mark J. Sarnak

**Affiliations:** 1 Division of Nephrology, Department of Medicine, Tufts Medical Center, 800 Washington Street, Box 391, Boston, MA 02111 USA; 2 Department of Neurobiology and Anatomy, Boston University School of Medicine, Boston, MA USA; 3 Department of Radiology, Beth-Israel Deaconess Medical Center, Boston, MA USA; 4 Mayo Clinic, Rochester, MN USA; 5 Division of Nephrology and Hypertension, Department of Internal Medicine, University of Kansas Medical Center, Kansas City, KS USA; 6 The Institute for Clinical Research and Health Policy Studies, Tufts Medical Center, Boston, MA USA; 7 Tufts Clinical and Translational Science Institute, Tufts University, Boston, MA USA; 8 Jean Mayer USDA Human Nutrition Research Center on Aging at Tufts University, Boston, MA USA

## Abstract

**Background:**

Patients treated with dialysis have high rates of brain infarcts, brain atrophy, and white matter disease. There are limited data regarding the presence of more subtle damage to brain white matter.

**Methods:**

In the Cognition and Dialysis Study, we compared brain structure using diffusion tensor imaging in hemodialysis (HD) patients to individuals without known kidney disease, using tract based spatial statistics (TBSS) to compare Fractional Anisotropy (FA) and Mean Diffusivity (MD). Statistical comparison of each overlaid voxel was age controlled using a permutation based corrected *p* value of <0.05.

**Results:**

Thirty-four HD patients and twenty six controls (52 vs 51 years for HD vs control) had adequate magnetic resonance imaging for analysis. The HD group had fewer women (38% vs 23%) and a higher prevalence of diabetes (29% vs 8%), heart failure (29% vs 0%) and clinical stroke (15% vs 0%). Hemodialysis patients had significantly lower FA across multiple white matter fiber tracts, with fronto-temporal connections, the genu of the corpus callosum and the fornix more significantly affected than posterior regions of the brain. Similarly, HD patients had significantly higher mean diffusivity in multiple anterior brain regions. Results remained similar when those with a prior history of stroke were excluded.

**Conclusions:**

In HD patients, there is more white matter disease in the anterior than posterior parts of the brain compared to controls without kidney disease. This pattern of injury is most similar to that seen in aging, suggesting that developing chronic kidney disease and ultimately kidney failure may result in a phenotype consistent with accelerated aging.

## Background

Anatomic brain abnormalities, including stroke and brain atrophy, are common in patients with kidney failure [1–5]. Patients receiving maintenance dialysis are also known to have a high burden of white matter disease [1, 5, 6], a finding closely correlated with incident clinical stroke [7–9]. White matter disease is associated with adverse outcomes in the general population and in CKD [10], including higher risk for overt strokes and cognitive impairment [8, 11].

Through measurement of water diffusivity in the brain, diffusion tensor imaging (DTI) allows for the characterization of alterations to white matter tracts in addition to localization of white matter damage to specific brain regions [12]. In those without kidney disease, DTI shows promise in detailing white matter disease related to stroke [13], aging, [14] Alzheimer’s disease, [15] and multiple sclerosis [16]. Although multiple prior studies have assessed the general burden of white matter disease in kidney disease patients using conventional MRI, very few have utilized DTI [17, 18]. Of those investigating DTI in dialysis patients, firm conclusions have been limited by homogeneous populations, the use of low resolution magnetic resonance imaging (MRI), and the lack of use of tract-based spatial statistics to improve the sensitivity and objectivity of DTI [19].

Dialysis patients are at particularly high risk for stroke [20] and cognitive impairment [21, 22], and white matter disease may be a precursor to each of these adverse outcomes; accordingly, the presence and location of DTI abnormalities in patients receiving dialysis may be of particular interest. We therefore obtained high resolution MR brain imaging in stable maintenance hemodialysis (HD) patients and in participants already scheduled for a brain MRI who had no reported history of kidney disease, utilizing DTI and tract-based spatial statistics to detect and localize damage to white matter bundle pathways.

## Methods

### Study population

All patients receiving hemodialysis at five Dialysis Clinic Inc. (DCI) units and one hospital-based dialysis unit in the greater Boston, MA area who enrolled at baseline in the Cognition and Dialysis Study [22] (01/21/04 to 06/29/11), a prospective cohort of maintenance hemodialysis patients, were approached to consent for brain MRI scans. Eligibility criteria for the Cognition and Dialysis Study is described elsewhere [22]. Briefly, eligibility criteria were age 18 years or older, English fluency, medically stable condition, and receipt of hemodialysis therapy for at least one month. The most common reasons for not undergoing an MRI were lack of interest and therefore not providing consent, or ineligibility for MRI due to metallic and electronic implants. Demographic information regarding dialysis vintage, etiology of end-stage renal disease, and cardiovascular disease risk factors including history of diabetes, hypertension, coronary artery disease, stroke, and congestive heart failure was patient-specific and obtained from patient history and individual patient chart review, including paper charts and dialysis and hospital electronic health records for each patient.

Controls were recruited from Tufts Medical Center (Tufts MC) and were approached if they were already scheduled for a brain MRI for another indication and were between 18 and 75 years of age. After obtaining the originally scheduled MRI scan, an additional scan to obtain DT images was performed. Among controls, the most common indications for undergoing a MRI were headache (56%), vertigo (8%), and facial pain (8%). Exclusion criteria were known kidney disease, a presentation with symptoms or signs of stroke or history of stroke, psychiatric disease, dementia, other serious neurological disorders and history of malignancy. Self-reported demographic data on age, sex, race, and history of diabetes, hypertension, coronary artery disease, and congestive heart failure were collected at time of enrollment. Confirmation of the absence of kidney disease was determined through documentation of estimated glomerular filtration rate (eGFR, CKD-EPI eq. [23]) of greater than 60 ml/min/ per 1.73 m^2^ within 1 year of MRI (80%) or through review of the medical record if an eGFR was not available (20%). The Tufts MC Institutional Review Board approved the study, and all participants signed informed consent allowing for review of individual medical records as well as participation in the study.

### Outcomes

Magnetic resonance imaging was performed on 45 participants in the hemodialysis group and 37 participants in the control group. Diffusion tensor imaging was performed on a 3***-***T Philips scanner using a single-shot, spin-echo, echo-planar sequence with 16 independent directions. Eleven subjects in the hemodialysis group and eleven subjects in the control group were excluded from analysis due to an inadequate MRI field of view, which is required for the normalization process to utilize tract based spatial statistics [19]. Thirty four DT images in the hemodialysis group and 26 DT images were determined to be suitable for subsequent analysis.

Diffusion tensor imaging allows for the measurement of water displacement within the brain, yielding two primary outcomes which provide information on white matter integrity: fractional anisotropy and mean diffusivity [12]. Fractional anisotropy is a measure of how quickly water diffuses across white matter fibers; normal white matter is associated with high FA levels indicating normal (fast) diffusion of water, while white matter damage results in slower diffusion of water [12]. As such, lower FA values indicate a loss of white matter integrity and are interpreted as white matter disease. Mean diffusivity is a summary measure of the molecular diffusion rate, which provides complementary information to FA, with higher values indicating either edema or white matter loss of integrity.

Diffusion tensor imaging processing was performed using the FSL software package (https://fsl.fmrib.ox.ac.uk/fsl/fslwiki). Raw DTI data was preprocessed for correcting eddy-currents and head motion using the diffusion toolbox (part of FSL). Then, fractional anisotropy (FA) and mean diffusivity (MD) were calculated and used for the TBSS processing. TBSS processing includes, 1) a creation of a standard brain template consisting of a three dimensional skeleton highlighting white matter fiber tracts, 2) spatial normalization of subject data into the standard space and 3) performing voxel-wise statistical comparisons along the major white matter pathways [19, 24].

### Statistical analysis

Demographic characteristics for the hemodialysis group and control group were reported as means with standard deviations or percentages and were compared using Fisher exact tests or *t* tests as appropriate. These analyses were performed using SAS software (version 9.3, SAS Institute, Cary NC) and all hypothesis tests were two-sided, with a *p* < 0.05 considered as significant. Voxel-wise comparison between the hemodialysis and control groups was performed using TBSS. All TBSS analyses were controlled for age and used a permutation-based inference method of nonparametric statistical thresholding; [24] this method addresses the problem of multiple comparisons by using a null distribution of the maximum expected voxel-wise test statistics. For this study, 10,000 iterations were used to calculate inferences, with a corrected *p* value of <0.05 considered significant.

To examine how cardiovascular disease and its risk factors within hemodialysis patients impact white matter integrity, we divided the hemodialysis group into two subgroups based on vascular disease history and risk factors (diabetes, coronary artery disease, stroke, or congestive heart failure). Group A includes those HD patients with two or more risk factors while Group B includes those with one or zero risk factors. Each subgroup was then compared to the control group without history of kidney disease.

### Sensitivity analyses

To examine how a prior history of clinical stroke impacted differences in white matter disease, we repeated analyses removing those HD patients with a prior reported history of stroke and compared them to the control group.

## Results

Thirty-four HD patients and twenty six controls had adequate imaging for analysis. The mean age was similar (52 years vs 51 years for HD vs control), while the HD group had fewer women (38% vs 23%), more participants who were black (38% vs 19%) and a higher rates of diabetes (29% vs 8%), heart failure (29% vs 0%), stroke (15% vs 0%) and hypertension (85% vs 35%) (Table 1). The median dialysis vintage (time since initiation of hemodialysis) was 18 months (7, 40 months for 25th and 75th percentiles, respectively). A variety of etiologies leading to end-stage renal disease was present, with diabetes and hypertension (32%), glomerulonephritis (40%), and hereditary kidney disease (12%) being the most prominent. Fifteen (44%) of the thirty-four HD patients had more than one cardiovascular disease risk factor.

**Table 1:** Clinical characteristics of hemodialysis and control groups

	Hemodialysis (*N* = 34)	Control (*N* = 26)	*P* value
Age - years (SD)	51.7 (17.3)	50.7 (16.7)	0.6
Female	38%	69%	0.02
African American	38%	19%	0.16
Diabetes	29%	8%	0.05
Stroke	15%	0%	0.06
Heart Failure	29%	0%	0.003
Coronary Artery Disease	15%	0%	0.06
Hypertension	85%	35%	<0.001

### Diffusion tensor imaging findings

All analyses were controlled for age. Results are reported predominantly as images to allow for appropriate visualization of specific anatomic brain regions through the voxel-wise analyses.

#### Fractional anisotropy

**Fig. 1:**
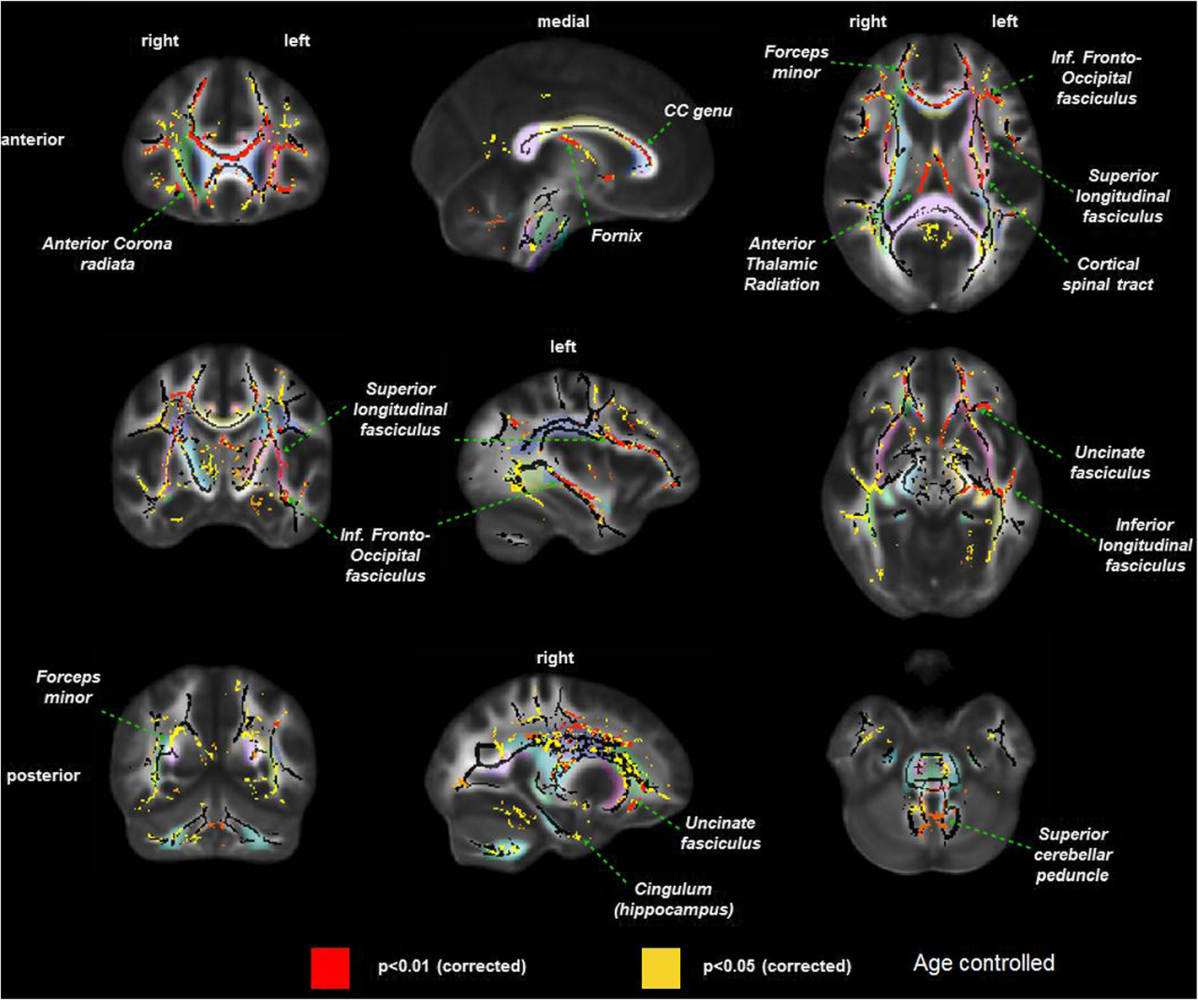
Difference in white matter damage assessed by fractional anisotropy in prevalent hemodialysis patients (*n* = 34) versus controls without kidney disease (*n* = 26), controlled for age. *p* values are corrected to account for multiple testing. *Left column* = coronal view, *middle column* = sagittal view, *right column* = axial view. *Red* = areas with most significant differences in FA (*p* < 0.01), *yellow* = remaining areas with differences in FA (*p* < 0.05). *Pastel colors* indicate distinct white matter fibers/bundles, which are labeled within the figure

Hemodialysis patients had significantly lower FA across multiple white matter fiber tracts compared to controls without kidney disease. Specifically, we observed large clusters of differences within the fronto-temporal connections, the genu of the corpus callosum and the fornix. In contrast, the posterior parts of the brain showed less difference in FA between HD patients and controls (Fig. 1).

#### Mean diffusivity

**Fig. 2:**
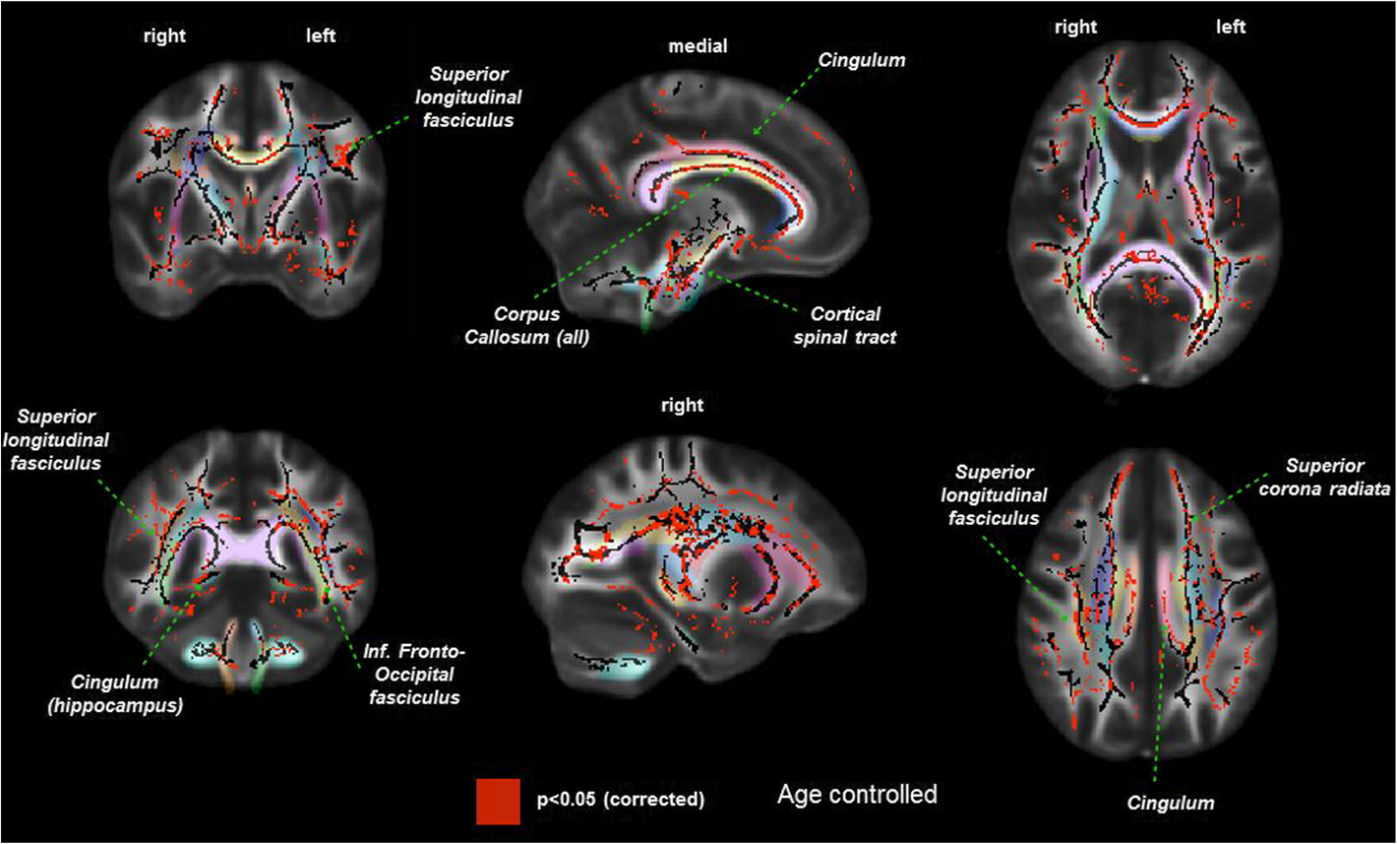
Difference in white matter damage assessed by mean diffusivity in prevalent hemodialysis patients (*n* = 34) versus controls without kidney disease (*n* = 26). *Left column* = coronal view, *middle column* = sagittal view, *right column* = axial view. *Red* = areas with significant differences in FA (*p* < 0.05). *Pastel colors* indicate distinct white matter fibers/bundles, which are labeled within the figure

Similar to the above FA results, the HD group had significantly higher mean diffusivity in multiple brain regions compared to controls (Fig. 2), though the difference between anterior and posterior appeared to be less pronounced than with FA.

#### Comparison between subgroups of HD patients and controls

**Fig. 3:**
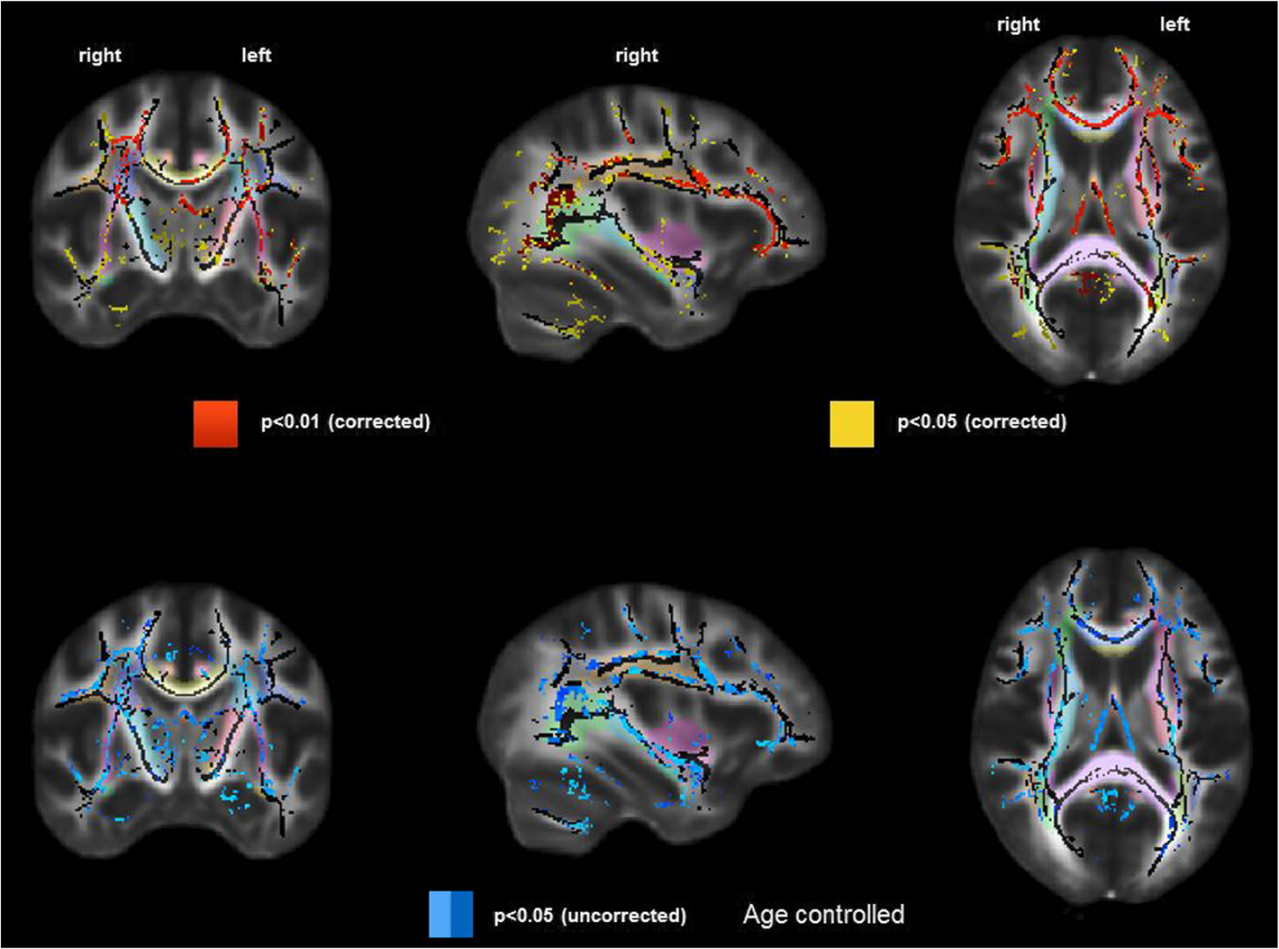
Difference in white matter damage assessed by fractional anisotropy in prevalent hemodialysis patients, separated by number of cardiovascular risk factors (zero or one vs more than one) compared to controls without kidney disease. *Top row* = Hemodialysis group with more than one vascular disease risk factor (*n* = 17) vs controls without kidney disease (*n* = 17). *Bottom row* = Hemodialysis group with zero or one vascular disease risk factor (*n* = 17) vs controls without kidney disease (*n* = 17). *Left column* = coronal view, *middle column* = sagittal view, *right column* = axial view. *Red* = areas with the most significant differences in FA, *yellow* = remaining areas with significant differences in FA. *Pastel colors* indicate distinct white matter fibers/bundles

HD patients with two or more risk factors showed substantial differences compared to controls in FA across multiple white fiber tracts with predominant anterior involvement (Fig. 3, upper panel). In contrast, those HD patients with one or no risk factors demonstrated no significant differences in FA values compared to controls (Fig. 3, lower panel).

## Discussion

Diffusion tensor imaging in prevalent HD patients demonstrated multiple brain areas with low FA and high MD as compared to a group of participants without kidney disease. These findings are consistent with a loss of white matter integrity, mainly clustered in the anterior of the brain (fronto-temporal connections, genu of corpus callosum and fornix). This pattern of injury seen within HD patients is most similar to that seen with aging [25, 26], despite the HD group having a mean age of 52 years. HD patients with more than one cardiovascular disease risk factors appeared to account for much of the difference in white matter integrity. However, we note that the primary goal of our manuscript is to highlight the significant difference in white matter disease between HD and controls, recognizing that there are many reasons, including comorbidity, for this difference and that we cannot fully adjust for all factors.

Multiple prior studies have demonstrated an increased prevalence and severity of white matter changes in both patients with chronic kidney disease as well as those with end-stage renal disease requiring dialysis [1, 5, 6]. However, these studies have typically relied on conventional MR imaging, which does not allow for direct voxel by voxel comparison of white matter integrity between patients and controls and is poorly suited for the identification of localized differences in white matter integrity.

There are currently limited and inconsistent data on diffusion tensor imaging in patients receiving hemodialysis. Three prior studies of DTI in a population of HD patients have been published. Chou et al. performed DTI in 28 HD patients and 25 age matched controls using a 1.5 T MR scanner, demonstrating increased MD and decreased FA in the HD group versus controls [17]. A study by Kong et al. obtained DTI in 80 HD patients and 80 controls, also showed decreased FA and increased MD for the HD group in comparison with controls [18]. Each of these studies, which were limited to Asian populations, reported widespread increased MD throughout the brain, without a clear pattern across different brain regions or tracts. A third study by McIntyre et al. reported the results of a clinical trial examining if the cooling of dialysate prevented white matter damage in prevalent hemodialysis patients [27]. Participants in the cooling arm (0.5 degrees C below core body temp) showed no change in DTI measures over one year, while those dialyzed at 37 °C demonstrated an *increase* in FA over one year. This result appears contrary to both our findings and those previously published, which generally have agreed that decreased FA is consistent white matter injury. Our study confirms that HD patients have decreased FA and increased MD compared to controls, but differs from these prior studies by demonstrating a predominantly frontal pattern of white matter injury. The strengths of our study include the use of high resolution 3 T MRI and use of TBSS analysis, a more diverse cohort than prior DTI studies, and ascertainment of cardiovascular risk factors. The inclusion of participants of differing races, as well as those with multiple cardiovascular disease risk factors may account for the spatial differences in white matter damage. In fact, a DTI study of ESRD patients in the United States who ultimately underwent renal transplantation also demonstrated anterior pattern of white matter damage, which showed improvement after transplantation [28]. An anterior pattern of white matter damage appears most similar to that seen when DTI is performed within healthy elderly patients [25, 26]. Limitations to our study include a relatively small number of participants, a younger age than the average U.S. hemodialysis patients, and the use of prevalent HD patients. Taken together, these limitations may limit our ability to generalize these findings to the overall dialysis population. In addition, our control group is overall healthier than the hemodialysis group, reflecting an absence of kidney disease as an inclusion criterion. A greater number and severity of comorbid conditions such as vascular disease in the hemodialysis cohort may be the primary reason for our findings.

There are several possible explanations for our findings. First, white matter damage/disease is reported to be a possible precursor for clinical vascular disease, with strong associations seen between the presence of white matter disease and future risk of transient ischemic attack or stroke [7, 9]. Patients with kidney failure have a high prevalence of vascular disease risk factors (diabetes, hypertension, etc.) and have an increased risk of developing vascular disease [20, 29] which together may predispose towards greater white matter damage. There likely are additional etiologies underlying our findings. Hemodialysis patients, in comparison with PD patients, may be at high risk for recurrent brain injury due to hemodynamic changes during the procedure [30]. However, this cannot explain all of the increased risk as there is also high prevalence of white matter disease seen within those with CKD [10] as well as ESRD patients receiving peritoneal dialysis, [6] neither of whom are subject to sudden hemodynamic shifts. Finally, there is the possibility that kidney failure or kidney disease itself may predispose to brain injury [31], perhaps through retention of harmful toxins (a uremia-like effect) or through dysregulation of existing hormonal pathways, such as with mineral metabolism.

The pattern of anterior white matter damage is most similar to that observed in studies of the effect of aging on white matter integrity [25, 26]. Prior MRI studies have also demonstrated that brain atrophy is common within dialysis patients [2, 5], a finding that is pronounced in elderly individuals [32]. Taken together, these observations may suggest that kidney disease accelerates or mimics the effect of aging on the brain. Alternatively, patients receiving dialysis, particularly those who developed kidney failure from diabetes and/or hypertension, often have developed co-morbid conditions such as vascular disease at a younger age than the general population. A prolonged effect of diabetes and hypertension upon the brain could account for the severity of disease observed among hemodialysis patients in our cohort, despite relatively young ages.

## Conclusions

Diffusion tensor imaging performed within a group of patients receiving hemodialysis versus controls without kidney disease demonstrated significant alterations in white matter integrity. Damage was primarily located within anterior brain regions, in contrast to posterior regions which were on average more similar to the control group. These findings may occur either due to a propensity for cerebrovascular disease, through hemodynamic effects mediated by hemodialysis, or be mediated by kidney failure itself. Future studies should focus on the pathophysiology and risk factors that may lead to white matter damage in kidney disease patients as well as methods to prevent or limit brain injury.

## Abbreviations

CKD: Chronic kidney disease
DTI: Diffusion tensor imaging
ESRD: End-stage renal disease
FA: Fractional anisotropy
HD: Hemodialysis
MD: Mean diffusivity
MRI: Magnetic resonance imaging
TBSS: Tract based spatial statistics
